# Survival of a Novel Subset of Midbrain Dopaminergic Neurons Projecting to the Lateral Septum Is Dependent on NeuroD Proteins

**DOI:** 10.1523/JNEUROSCI.2414-16.2016

**Published:** 2017-03-01

**Authors:** Shabana Khan, Simon R.W. Stott, Audrey Chabrat, Anna M. Truckenbrodt, Bradley Spencer-Dene, Klaus-Armin Nave, François Guillemot, Martin Levesque, Siew-Lan Ang

**Affiliations:** ^1^Francis Crick Institute, Mill Hill Laboratory, Mill Hill, NW7 1AA, United Kingdom,; ^2^Department of Psychiatry and Neurosciences, Université Laval, Quebec G1V 0A6, Canada,; ^3^Centre de recherche de l'Institut universitaire en santé mentale de Québec, Quebec G1J 2G3, Canada,; ^4^Francis Crick Institute, Lincoln's Inn Fields Laboratory, London, WC2A 3LY, United Kingdom, and; ^5^Department of Neurogenetics, Max Planck Institute of Experimental Medicine, 37075 Göttingen, Germany

**Keywords:** lateral septum, midbrain dopaminergic neurons, Neurod1, Neurod6, neuronal diversity, ventral tegmental area

## Abstract

Midbrain dopaminergic neurons are highly heterogeneous. They differ in their connectivity and firing patterns and, therefore, in their functional properties. The molecular underpinnings of this heterogeneity are largely unknown, and there is a paucity of markers that distinguish these functional subsets. In this paper, we report the identification and characterization of a novel subset of midbrain dopaminergic neurons located in the ventral tegmental area that expresses the basic helix-loop-helix transcription factor, Neurogenic Differentiation Factor-6 (NEUROD6). Retrograde fluorogold tracing experiments demonstrate that *Neurod6*^+^ midbrain dopaminergic neurons neurons project to two distinct septal regions: the dorsal and intermediate region of the lateral septum. Loss-of-function studies in mice demonstrate that *Neurod6* and the closely related family member *Neurod1* are both specifically required for the survival of this lateral-septum projecting neuronal subset during development. Our findings underscore the complex organization of midbrain dopaminergic neurons and provide an entry point for future studies of the functions of the *Neurod6*^+^ subset of midbrain dopaminergic neurons.

**SIGNIFICANCE STATEMENT** Midbrain dopaminergic neurons regulate diverse brain functions, including voluntary movement and cognitive and emotive behaviors. These neurons are heterogeneous, and distinct subsets are thought to regulate different behaviors. However, we currently lack the means to identify and modify gene function in specific subsets of midbrain dopaminergic neurons. In this study, we identify the transcription factor NEUROD6 as a specific marker for a novel subset of midbrain dopaminergic neurons in the ventral midbrain that project to the lateral septum, and we reveal essential roles for *Neurod1* and *Neurod6* in the survival of these neurons during development. Our findings highlight the molecular and anatomical heterogeneity of midbrain dopaminergic neurons and contribute to a better understanding of this functionally complex group of neurons.

## Introduction

Midbrain dopaminergic (mDA) neurons are mostly found in the substantia nigra pars compacta (SNc) and ventral tegmental area (VTA) and regulate multiple brain functions, including voluntary movement, working memory, emotion, and cognition (Björklund and Dunnett, 2007). These neurons project to the forebrain and were initially thought of as a homogeneous group of neurons based on their common use of dopamine as a neurotransmitter for intercellular communication. However, it is now becoming clear that mDA neurons are heterogeneous in regard to their target and afferent projections (Roeper, 2013; Beier et al., 2015; Menegas et al., 2015), firing patterns (Roeper, 2013), and gene expression profiles (Poulin et al., 2014), all of which impact on their functional properties. mDA neurons projecting to striatal spiny projection neurons in the nucleus accumbens medial shell use glutamate as a cotransmitter (Hnasko et al., 2010; Stuber et al., 2010), whereas those projecting to the dorsal striatum use GABA (Tritsch et al., 2012). Furthermore, intact-brain analyses, using a combination of whole-brain imaging, optogenetics, viral tracing, and fiber photometry, have revealed that different subsets of SNc neurons contribute to different nigrostriatal circuits carrying different information streams (Lerner et al., 2015).

Despite the emerging evidence for functionally distinct subsets of mDA neurons, we still know little of the molecular underpinnings of this functional diversity. Recent transcriptome analyses of VTA and SNc mDA neurons have provided lists of genes differentially expressed between these two anatomically separable groups of neurons (Grimm et al., 2004; Chung et al., 2005). One of these differentially expressed genes is the basic helix-loop-helix (bHLH) transcription factor NEUROD6, alternatively known as NEX1, MATH2, and ATOH2. NEUROD6 belongs to the NEUROD subfamily of basic-helix-loop (bHLH) transcription factors, which consists of 4 members, including NEUROD1, NEUROD2, NEUROD4, and NEUROD6 (Bertrand et al., 2002). NEUROD2 and NEUROD6 are both required for the fasciculation and directional growth of callosal axons in the mouse neocortex (Bormuth et al., 2013). NEUROD6 also specifies the fate of a subtype of retinal amacrine cells (Cherry et al., 2011; Kay et al., 2011), and it has been implicated in the survival of cultured rat pheochromocytoma PC12 cells, where it enhances mitochondrial biogenesis and regulates cytoskeletal organization (Uittenbogaard and Chiaramello, 2005; Uittenbogaard et al., 2010; Baxter et al., 2012). These important roles of NEUROD6 in neuronal differentiation and survival raised the possibility of similar roles for this factor in mDA neurons.

We have therefore investigated the role of NEUROD6 in the development of mDA neurons using gene expression analysis and retrograde Fluorogold (FG) tracing experiments in wild-type and *Neurod6*-null mutant mice (Goebbels et al., 2006). Our findings revealed that *Neurod6* is specifically expressed in a subset of mDA neurons in the VTA that project to the intermediate (LSi) and dorsal regions (LSd) of the lateral septum. *Neurod6* alone is required for the survival of LSi-projecting mDA neurons; however, some *Neurod6*^+^ neurons still develop normally in *Neurod6* mutant mice and send axons to the LSd. Given this partial loss of *Neurod6*^+^ mDA neurons, we also analyzed *Neurod6* and *Neurod1* double-mutant embryos and found that *Neurod1* also regulates the survival of the *Neurod6*^+^ mDA neurons. This study has identified a novel subset of mDA neurons that projects to the lateral septum and has a unique molecular signature. Our results also demonstrate essential roles for *Neurod1* and *Neurod6* in this subset of mDA neurons during development.

## Materials and Methods

### 

#### 

##### Generation and genotyping of mutant embryos and animals.

*Neurod6-Cre* is a knock-in mouse line that expresses Cre recombinase under the control of *Neurod6* regulatory sequences (Goebbels et al., 2006). We first generated *Neurod6*^*Cre*/+^;*R26R^YFP/YFP^* mice (referred to henceforth as *Neurod6* control mice) by sequential breedings of Neurod6^Cre/+^ animals (Goebbels et al., 2006) with *R26R^YFP/YFP^* reporter mice. We intercrossed control *Neurod6*^*Cre*/+^;*R26R^YFP/YFP^* mice to generate *Neurod6^Cre/Cre^;R26R^YFP/YFP^* single-homozygous mutant embryos and mice. For double-mutant studies, *Neurod1*^*LacZ*/+^*;Neurod6*^*Cre*/+^*;R26R^YFP/YFP^* mice were generated by breeding *Neurod1*^*LacZ*/+^ mice (Miyata et al., 1999) with *Neurod6^Cre/Cre^;R26R^YFP/YFP^* animals. These animals were then intercrossed to generate double-homozygous mutant embryos and mice carrying different numbers of *Neurod1* and *Neurod6* mutant alleles. *Neurod6* and *Neurod1* heterozygous and homozygous allelic deletions were determined by PCR as described previously (Schwab et al., 2000; Chao et al., 2007). All male and female animals used were from mixed background. At all times, animals were handled according to the Society of Neuroscience policy on the use of animals in Neuroscience research, as well as the European Communities Council Directive.

##### Tissue preparation.

For *in situ* hybridization and immunohistochemistry experiments, 2-month-old adult animals were deeply anesthetized with ketamine-xylazine (10 mg/ml and 1 mg/ml, respectively) and were transcardially perfused with PBS, pH 7.4, followed by 4% (w/v) formaldehyde in PBS, pH 7.4. Embryonic, postnatal, and adult brains were removed, immersion-fixed in fixative 4% (w/v) formaldehyde in 100 mm phosphate buffer, pH 7.4, overnight at 4°C, and subsequently cryoprotected in 30% (w/v) sucrose-PBS. Tissue samples were embedded in optimum cutting temperature compound (VWR International) and sectioned on a cryostat (CM3050S; Leica) as 20 μm sections on Superfrost Plus microscope slides (25 × 75 × 1.0 mm; Thermo Scientific) for embryonic brains and 35–50 μm free-floating sections for postnatal and adult brains.

For RNAscope experiments, adult animals were deeply anesthetized with ketamine-xylazine (10 mg/ml and 1 mg/ml, respectively) and were transcardially perfused with 10% neutral buffered formalin (Sigma-Aldrich). Brains were then removed and immersion-fixed in 10% neutral buffered formalin overnight at room temperature and then transferred into 70% ethanol for storage. Brains were subsequently embedded in paraffin wax and processed into 4 μm sections collected onto Superfrost Plus microscope slides (25 × 75 × 1.0 mm; Thermo Scientific) using the Leica RM2255 microtome.

##### *In situ* hybridization.

Section *in situ* hybridizations were performed as previously described (Vernay et al., 2005) or using the RNAscope 2.0 HD Brown Chromogenic Reagent Kit according to the manufacturer's instructions (Advanced Cell Diagnostics). The following mouse antisense RNA probes have been used: *Neurod6* (Bröhl et al., 2008), *Neurod1* (Lee et al., 1995), and *TH* (Grima et al., 1985). For RNAscope experiments, target probes for *Neurod6* and *Grp* were designed by Advanced Cell Diagnostics. For each probe, a minimum of three control and three mutant brains were analyzed at embryonic and adult stages.

##### Immunohistochemistry.

For immunohistochemistry, sections were incubated overnight at 4°C with the appropriate primary antibody diluted in 1% (w/v) BSA in PBS with 0.1% Triton X-100. Sections were washed thoroughly and subsequently incubated for 1 h at room temperature with a secondary antibody conjugated with a fluorochrome (Invitrogen) diluted in 1% BSA in 100 mm PBS. For nuclear staining, sections were incubated with DAPI. Sections were then washed extensively overnight in 100 mm PBS and mounted in Vectashield H-1000 (Vector Laboratories).

The following primary antibodies were used: sheep anti-GFP (1:1000; Bio-Rad/AbD Serotec), rabbit anti-TH (1:1000; Pelfreez), mouse anti-TH (1:500; Immunostar), sheep anti-TH (1:1000; Millipore), rabbit anti-OTX2 (1:500; Baas et al., 2000), goat anti-OTX2 (1:500; R&D Systems), mouse anti-CALBINDIN1 (1:1000; Swant), rabbit anti-ALDH1A1 (1:200; Abcam), mouse anti-NEUROD1 (1:500; Abcam), rabbit anti-NEUROD2 (1:1000; Abcam), and rabbit anti-TOM20 (1:1000; Santa Cruz Biotechnology). The following secondary antibodies were used: AlexaFluor-594 donkey anti-rabbit (1:300; Invitrogen), AlexaFluor-488 donkey anti-sheep (1:200; Invitrogen), FITC donkey anti-sheep (1:200; Jackson ImmunoResearch Laboratories), AlexaFluor-647 donkey anti-mouse (1:200; Invitrogen), Cy5 donkey anti-mouse (1:200; Jackson ImmunoResearch Laboratories), and Cy5 donkey anti-sheep (1:200; Jackson ImmunoResearch Laboratories).

##### Cell counting and imaging.

For each brain, 50 μm free-floating coronal cryosections were collected from the caudal to the rostral midbrain for immunohistochemistry with TH and GFP primary antibodies. Free-floating sections were subsequently mounted onto slides from caudal to the rostral midbrain and imaged using the Olympus virtual Slide microscope VS120-L100-W, Zeiss Apotome.2 microscope, and Leica TCS SP5 confocal microscope. YFP^+^/TH^+^ double-positive cells were counted for one hemisphere of all midbrain sections to determine both the (1) total cell number and (2) spatial distribution in controls and mutants at E18.5, P3, P7, P14, and adult stage. For each stage, three control and three mutant brains were analyzed. ImageJ (National Institutes of Health) software was used for cell quantification.

##### Statistical analysis.

Statistical analysis was performed only on cell counts of sections from the central mDA region. All sections through the central mDA region (bregma from −3.28 to −3.80 mm) contained 3 VTA nuclei, the paranigral nucleus (PN) and parabrachial nucleus (PBP), and the posterior portion of the interfascicular nucleus (IF) (Oades and Halliday, 1987), SNc as well as two adjacent nuclei, the interpeduncular nucleus, and the reticular magnocellular nuclei of the red nucleus (see Fig. 3*I*). Sections were matched according to the presence of these landmarks from the caudal to the rostral extent. In addition to these anatomical features, the most rostral section of the central mDA region also has the emergence of the fasciculus retroflexus. For cell count comparisons between two groups, statistical significance was assessed using the unpaired Student's *t* tests to determine differences in both the distribution and total number of TH^+^/YFP^+^ cells of the VTA in *Neurod6* controls and *Neurod6* mutants. When comparing cell number differences between more than two groups (*Neurod6* control, *Neurod6* mutant, and *Neurod1;Neurod6* double mutants), statistical significance was assessed by one-way ANOVA (GraphPad Prism) with Tukey's post test and comparing the means of each group to the mean of every other group.

##### Assay for cell apoptosis.

To detect cell apoptosis, TUNEL assays were performed using the TACS 2 TdT DAB *In Situ* Apoptosis Detection Kit (Trevigen, R&D Systems; 4810–30-K). TUNEL staining was performed according to the manufacturer's instructions. Once TUNEL was completed, sections were then analyzed for TH expression by immunohistochemistry.

##### Densitometry.

For the quantification of fluorescent intensity, images were acquired in structured illumination mode to achieve optical sectioning. Signal intensity of mitochondrial TOM-20 levels in YFP^+^/TH^+^ and YFP^−^/TH^+^ cells was quantified by Fiji image processing package. Regions of interest were accurately drawn around individual TH^+^ (YFP^+^ and YFP^−^) cells, and readings of fluorescence intensity were measured only from the TOM-20 channel. Only mDA neurons showing the nucleus in the optic sections were included in the analysis (>200 neurons were analyzed per cell type per genotype, from at least 5 pictures per group), and background fluorescence values were subtracted from all the readings.

##### Retrograde FG axonal labeling.

The retrograde tracer FG (Millipore) was injected into the lateral septum of *Neurod6*^*Cre*/+^;*R26R^YFP/YFP^* and *Neurod6^Cre/Cre^;R26R^YFP/YFP^* pups at postnatal day 10 (P10). Mice were anesthetized using isoflurane, and 1 μl of FG (2% in 0.9% sodium chloride and 2% biotin dextran amine [BDA], Invitrogen) was injected in both hemispheres. We adapted the stereotaxic coordinates of the atlas of Developing Mouse Brain (Paxinos and Watson, 2007), and the glass microsyringe was placed in the brain with the tip at 0.3 mm anterior to bregma, 0.2 mm lateral of the midline, and 1.8 mm from the surface of the brain. Mice were killed 3 d after injection for further analysis. Location of the injection sites was confirmed by staining the section with streptavidin-conjugated to AlexaFluor-568 that binds to BDA.

## Results

### Neurod6 identifies a subset of OTX2^+^, ALDH1A1^+^, CALBINDIN1^+^ mDA neurons in the VTA

**Figure 1.**
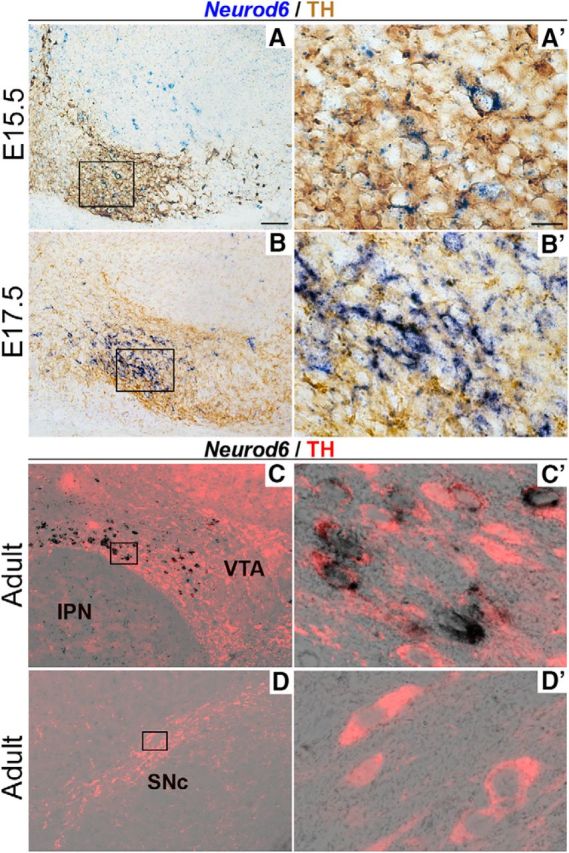
Selective expression of *Neurod6* in mDA neurons in the VTA. ***A–D***, *In situ* hybridization for *Neurod6* combined with immunohistochemistry for TH showing that *Neurod6* expression is restricted to TH^+^ mDA neurons located in ventral regions of the VTA from E15.5 to adult stage (***A–C***). In contrast, *Neurod6* is not expressed in TH^+^ mDA neurons in the SNc (***D***). ***A′–D′***, Higher magnification of boxed regions in corresponding panels ***A–D***. IPN, interpeduncular nucleus. Scale bars: ***A***, 200 μm; ***A′***, 10 μm.

We first examined the expression of *Neurod6* in the mouse midbrain by RNA *in situ* hybridization from embryonic days 12.5 (E12.5) onwards. *Neurod6* expression was not detected at E12.5 (data not shown), but transcripts were found localized in the ventral regions of the VTA at E15.5 (Fig. 1*A*,*A′*) and E17.5 (Fig. 1*B*,*B′*). To determine whether *Neurod6* is expressed in mDA neurons, immunohistochemistry for TH protein expression was conducted following detection of *Neurod6* transcripts. *Neurod6* transcripts were colocalized with TH protein in the VTA at embryonic stages (Fig. 1*A,A′*,*B*,*B′*, brown staining), and its expression was maintained in a subset of ventral VTA TH^+^ neurons in adult mice (Fig. 1*C–D′*). In contrast, *Neurod6* was not detected in TH^+^ neurons of the SNc at any stage (Fig. 1*C–D′*; and data not shown). Together, these results demonstrate that *Neurod6* is specifically expressed in a small subset of mDA neurons in the VTA from E15.5 to adulthood.

**Figure 2.**
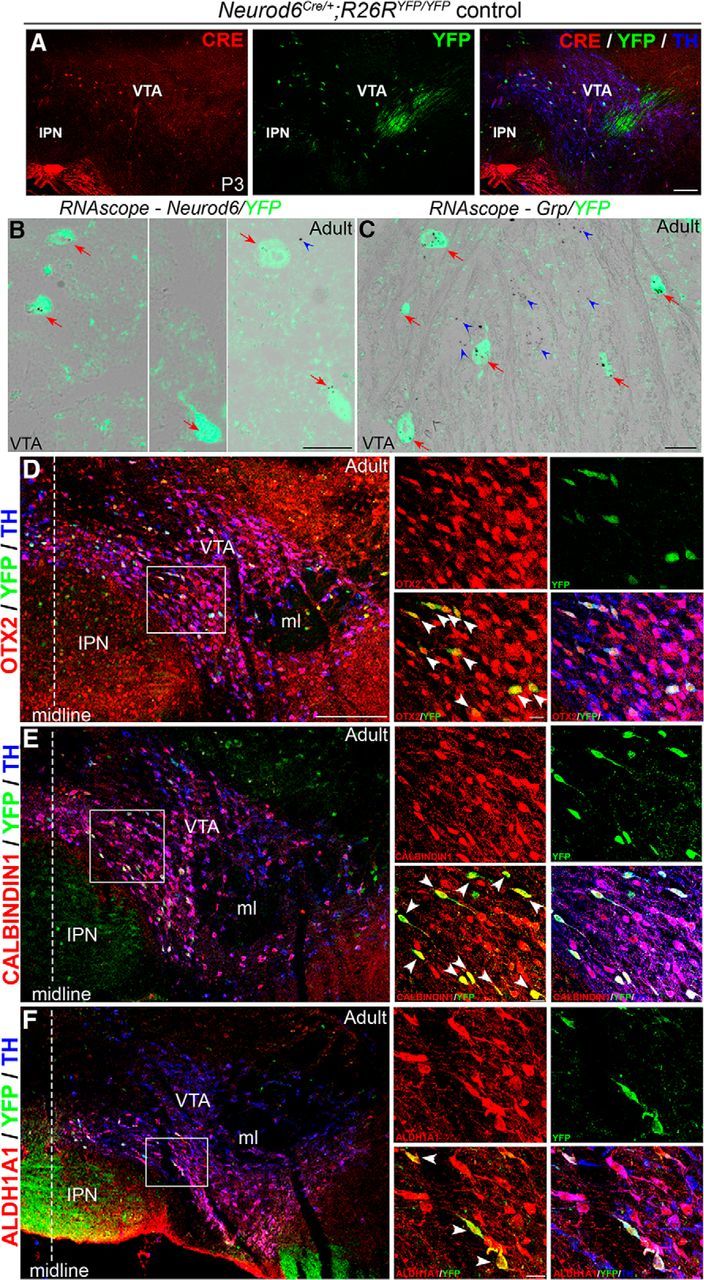
Identification of a novel subset of mDA neurons in the VTA that expresses *Neurod6*, OTX2, CALBINDIN1, ALDH1A1, and *Grp*. ***A***, Double antibody labeling showing that CRE is coexpressed with YFP in TH^+^ mDA neurons in the VTA of *Neurod6*^*Cre*/+^;*R26R^YFP/YFP^* pups at P3. ***B***, *In situ* hybridization of *Neurod6* combined with immunohistochemistry for YFP showing that all YFP^+^ cells express *Neurod6* transcripts (red arrows) in adult *Neurod6*^*Cre*/+^;*R26R^YFP/YFP^* mice. A few YFP^−^ cells also express *Neurod6* transcripts (blue arrowheads). ***C***, *In situ* hybridization of *Grp* combined with immunohistochemistry for YFP showing that *Grp* transcripts are detected in both YFP^+^ (red arrows) and YFP^−^ (blue arrowheads) cells in the VTA of adult *Neurod6*^*Cre*/+^ mice. ***D–F***, Triple antibody labeling showing that all YFP^+^/TH^+^ mDA neurons express OTX2 (***D***), CALBINDIN1 (***E***), and ALDH1A1 (***F***) in the VTA of adult *Neurod6*^*Cre*/+^;*R26R^YFP/YFP^* mice. IPN, Interpeduncular nucleus; ml, medial lemniscus. Dotted vertical lines indicate the midline of the section. White arrowheads indicate triple-labeled cells observed in the red and green channels only. Scale bars: ***A***, 100 μm; ***B***, ***C***, 200 μm (higher magnifications in ***D–F***); ***D–F***, 20 μm.

To facilitate colocalization studies between *Neurod6* and other genes expressed in mDA neurons in the VTA, and because a NEUROD6-specific antibody is not available, we used a mouse line where the coding region of *Neurod6* has been replaced by the *Cre* recombinase gene (Goebbels et al., 2006). We permanently labeled the cells in which the *Neurod6* promoter has been active by breeding these *Neurod6^Cre^* mice to *R26R^YFP^* reporter mice that conditionally express YFP in a Cre-dependent manner. We first confirmed that the CRE recombinase protein is coexpressed with YFP in VTA TH^+^ mDA neurons of *Neurod6*^*Cre*/+^*; R26R^YFP/YFP^* mice at P3 (Fig. 2*A*). We then showed that YFP expression is similar to endogenous *Neurod6* expression in the VTA of heterozygous *Neurod6*^*Cre*/+^*;R26R^YFP/YFP^* adult mice by conducting multiplex *in situ* hybridization using RNAscope followed by GFP antibody labeling. Indeed, all YFP^+^ neurons expressed *Neurod6* in the adult VTA (Fig. 2*B*). Together, these results establish that YFP expression in *Neurod6*^*Cre*/+^*;R26R^YFP/YFP^* mice faithfully reflects endogenous *Neurod6* expression and that expression of *Neurod6* is maintained in neurons once it has been initiated. We therefore used YFP expression in these mice as a marker of *Neurod6*^+^ mDA neurons in subsequent experiments.

Earlier studies have shown that expression of the homeodomain-containing transcription factor orthodenticle homeobox 2 (Otx2) in the midbrain is restricted to mDA neurons in the VTA (Di Salvio et al., 2010a, b). Moreover, mDA neurons in the VTA can be further subdivided into dorsal, central, and ventral layers based on coexpression of OTX2 with other markers, including CALBINDIN1 and aldehyde dehydrogenase 1a1 (ALDH1A1) (Di Salvio et al., 2010a). We therefore performed triple-antibody labeling experiments for TH, YFP, and these three proteins individually, to compare their expression patterns with that of *Neurod6* in mDA neurons. Our results show that all *Neurod6*^+^ cells express OTX2 (Fig. 2*D*), CALBINDIN1 (Fig. 2*E*), and ALDH1A1 (Fig. 2*F*), indicating the existence of a group of VTA neurons coexpressing all four genes. We also compared the expression of YFP and another VTA-enriched gene, gastrin releasing peptide (GRP; Paul Allen Brain Atlas) (Chung et al., 2005). RNAscope experiments using a *Grp* probe followed by antibody staining for GFP in *Neurod6*^*Cre*/+^*;R26R^YFP/YFP^* adult mice revealed that *Grp* was expressed in all *Neurod6*^+^ mDA neurons as well as in some surrounding *YFP*^−^ cells (Fig. 2*C*). In summary, our expression studies have identified a novel subset of VTA mDA neurons that coexpress *Neurod6*, OTX2, CALBINDIN1, ALDH1A1, and *Grp*.

### Neurod6 is required for the survival of a subset of VTA mDA neurons

**Figure 3.**
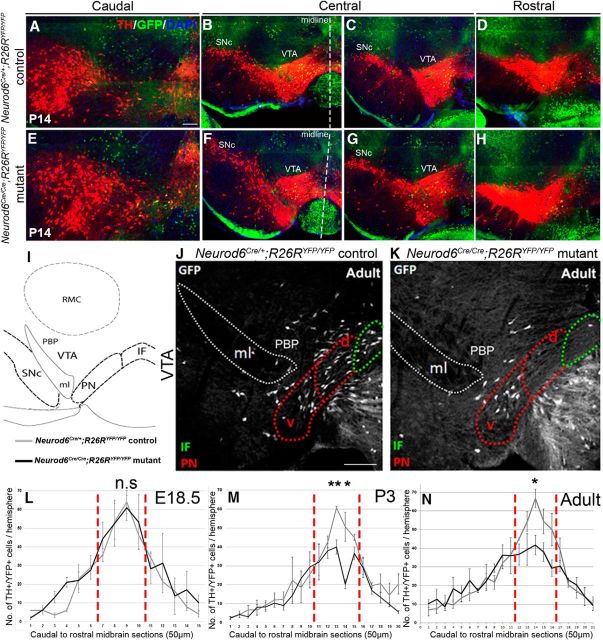
Partial reduction in the number of *Neurod6*^+^ mDA neurons in the absence of NEUROD6 function. ***A–H***, Immunohistochemistry for both YFP and TH on coronal sections from the caudal to the rostral extent of the mDA region at P14. Reduced numbers of YFP^+^/TH^+^ neurons are observed in the central mDA region (***B***, ***C***, ***F***, ***G***), whereas there is no apparent change in the numbers of YFP^+^/TH^+^ mDA neurons in the rostral and caudal midbrain at P14. ***I***, Schematic diagram showing the positions of mDA nuclei in the VTA and anatomical landmarks. ***J***, ***K***, Double antibody labeling of YFP and TH on a section through the central mDA region shows that YFP^+^/TH^+^ neurons are lost mostly in the dorsal region of the PN red dotted, PBP, and IF green dotted nuclei (only the YFP channel is shown). ***L–N***, Graph showing the number of YFP^+^/TH^+^ mDA neurons analyzed by immunohistochemistry on coronal midbrain sections from the caudal to the rostral extent of the mDA region at different stages. *Neurod6*^+^ mDA neurons in the VTA are lost predominantly in the central mDA region corresponding to sections demarcated by red vertical lines. RMC, Reticular magnocellular nuclei of the red nucleus; ml, medial lemniscus; d, dorsal; v, ventral; n.s., not significant. Error bars indicate SEM. *p < 0.05 (Student's *t* test). **p < 0.01 (Student's *t* test). Dotted vertical lines indicate the midline of the section. Scale bars: ***A***, 200 μm; ***J***, 100 μm.

To determine the role of *Neurod6* in mDA neurons, we analyzed *Neurod6^Cre/Cre^; R26R^YFP/YFP^* homozygous mutant mice. The total number and spatial distribution of *Neurod6*^+^ mDA neurons along the rostral-caudal axis of the VTA were evaluated by immunohistochemistry for TH and YFP at E18.5, postnatal day 3 (P3), P14, and 2 months (Fig. 3*A–H*; and data not shown). We focused our quantitative analysis on anatomically defined sections in the central mDA region (see Materials and Methods), which contain the highest numbers of *Neurod6*^+^ cells (i.e., 48% of the total number of VTA *Neurod6*^+^ mDA neurons, 1136 ± 82; Fig. 3*L–N*) and displayed a loss of *Neurod6*^+^ mDA neurons. At E18.5, we did not observe a significant difference in the total numbers of YFP^+^/TH^+^ cell in the central mDA region between *Neurod6* mutant and control embryos (Table 1). At postnatal stages, in contrast, there was a significant reduction in numbers of TH^+^/YFP^+^ mDA neurons in this region of *Neurod6* mutant compared with control mice (28% of control numbers at P3, 31% at P14, and 32% at 2 months; Fig. 3; Table 1). The VTA in adult mice can be further subdivided into several nuclei, including the IF, dorsal (dPN) and ventral paranigral nucleus (vPN), and PBP (Oades and Halliday, 1987). YFP^+^/TH^+^ cells were found in all these nuclei in wild-type mice (Fig. 3*J*) and were missing specifically in the IF, dPN, and PBP in *Neurod6* mutant mice (Fig. 3*K*). Together, these results show that the deletion of *Neurod6* results in a loss of ∼30% *Neurod6*^+^, TH^+^ mDA neurons in the central mDA region between E18.5 and P3 that persists at adult stages. YFP^+^/TH^+^ mDA neurons that subsist in the absence of *Neurod6* are localized in the vPN and the PBP (Fig. 3*K*).

**Table 1.** Loss of YFP^+^/TH^+^ cells in the central mDA region of *Neurod6^Cre/Cre^;R26R^YFP/YFP^* mutants per brain hemisphere*^a^*

	E18.5	P3	P14	Adult
*n*	3	3	3	3
*Neurod6* controls (mean ± SEM)	200 ± 3	232 ± 5	207 ± 1	270 ± 16
*Neurod6* mutants (mean ± SEM)	211 ± 27	167 ± 10	143 ± 14	185 ± 26
% of YFP^+^/TH^+^ mDA cells lost in mutants relative to controls	NA	28	31	32
Average no. of cells lost in mutants (mean ± SEM)	NA	65 ± 14	65 ± 14	85 ± 15
Significance	NS	**	**	*
*p* value	0.69	0.004	0.001	0.05

*^a^*Raw data of the numbers of mDA neurons counted per brain hemisphere in the central mDA region and the results from statistical analysis, comparing cell counts between *Neurod6* mutant and control brains at each stage using the unpaired Student's *t* test. *n*, Number of brain samples analyzed; NA, not applicable; NS, not significant.

*p < 0.05 (Student's *t* test);

**p < 0.005 (Student's *t* test).

**Figure 4.**
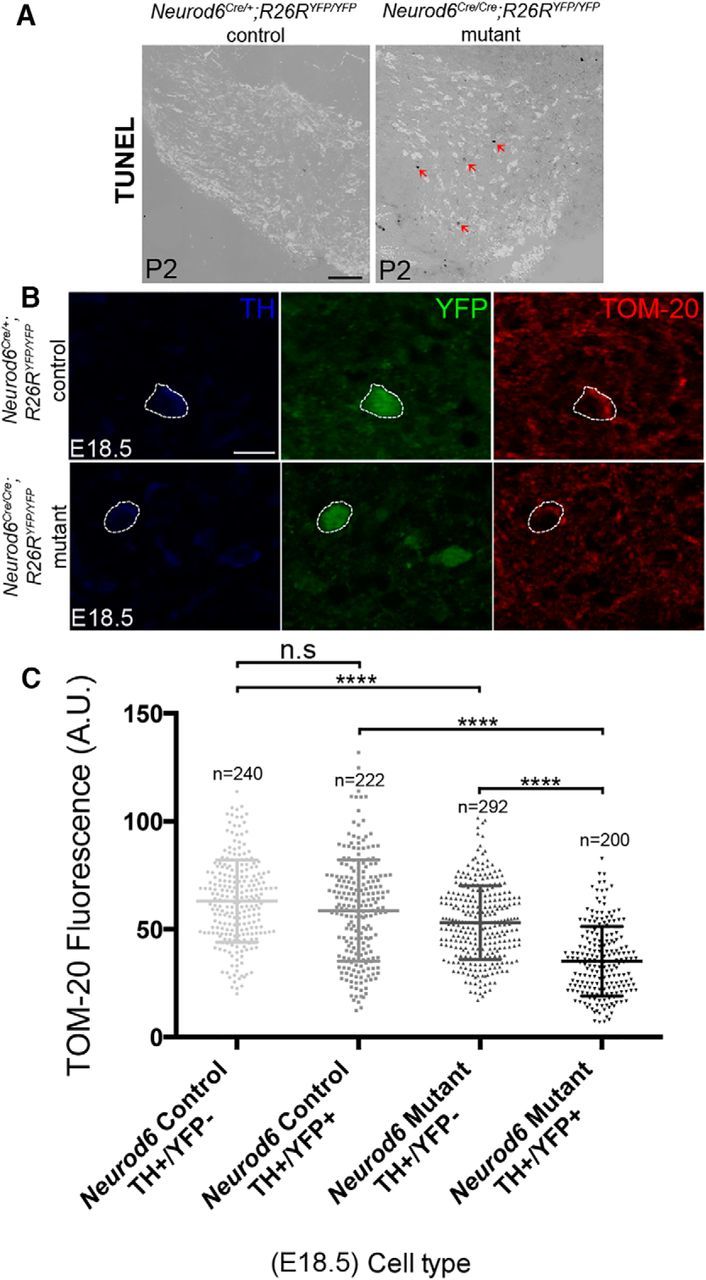
Loss of NEUROD6 function results in cell death and reduced mitochondrial mass in mDA neurons. ***A***, Apoptotic cells (arrowheads) revealed by TUNEL analysis are observed in the TH^+^ mDA region of *Neurod6* mutant, but not in control pups at P2. ***B***, A representative mDA neuron immunolabeled for TH, YFP, and TOM-20 that was used in quantitative analysis of mitochondrial mass. Dotted lines indicate cell boundary. ***C***, Reduction in mitochondrial mass measured by the mean fluorescence (normalized to cell area) of neurons analyzed by immunohistochemistry with TOM20, TH, and YFP (A.U., arbitrary units) in *Neurod6* control and mutant embryos at E18.5. *n*, Number of neurons analyzed. ****p < 0.0001 (one-way ANOVA, with Tukey's post test). Scale bars: ***A***, 200 μm; ***B***, 25 μm.

Next, we asked whether the absence of *Neurod6*^+^ neurons in *Neurod6* mutants from P3 onwards was due to apoptosis, and we defined the precise timing of elimination of these cells by conducting TUNEL analysis between E18.5 and P3. Some cells in the VTA of *Neurod6* mutant mice at P2 were TUNEL^+^ and expressed TH, although TUNEL^+^ cells were very rarely observed in control mice at this stage (Fig. 4*A*), indicating that *Neurod6*^+^ VTA neurons die from apoptosis at early postnatal stages in the absence of *Neurod6*.

The mechanisms of *Neurod6*-mediated neuronal survival have been extensively studied in PC12 cells where *Neurod6* functions to protect against oxidative stress by sustaining mitochondrial mass (Uittenbogaard et al., 2010). To assess changes in mitochondrial mass before neuronal cell death in *Neurod6* mutant VTA neurons, we examined the expression of the mitochondrial import receptor subunit (TOM-20) at E18.5. TOM-20 expression level in YFP^+^/TH^+^ VTA neurons, measured by densitometric analysis, was significantly decreased in *Neurod6* mutant compared with control embryo (Fig. 4*B*). A smaller but significant reduction in TOM-20 labeling was also observed in YFP- (*Neurod6*^−^)/TH^+^ mDA neurons (Fig. 4*B*). These results indicate a robust reduction in mitochondrial mass in both *Neurod6*^+^ and *Neurod6*^−^ mDA neurons, and the difference between these two neuronal populations is also significant. Therefore, the sustenance of a subset of VTA neurons by *Neurod6* is likely to involve the maintenance of their mitochondrial mass.

### Specific changes in axon projections of mDA neurons to the lateral septum in adult Neurod6 mutant mice

**Figure 5.**
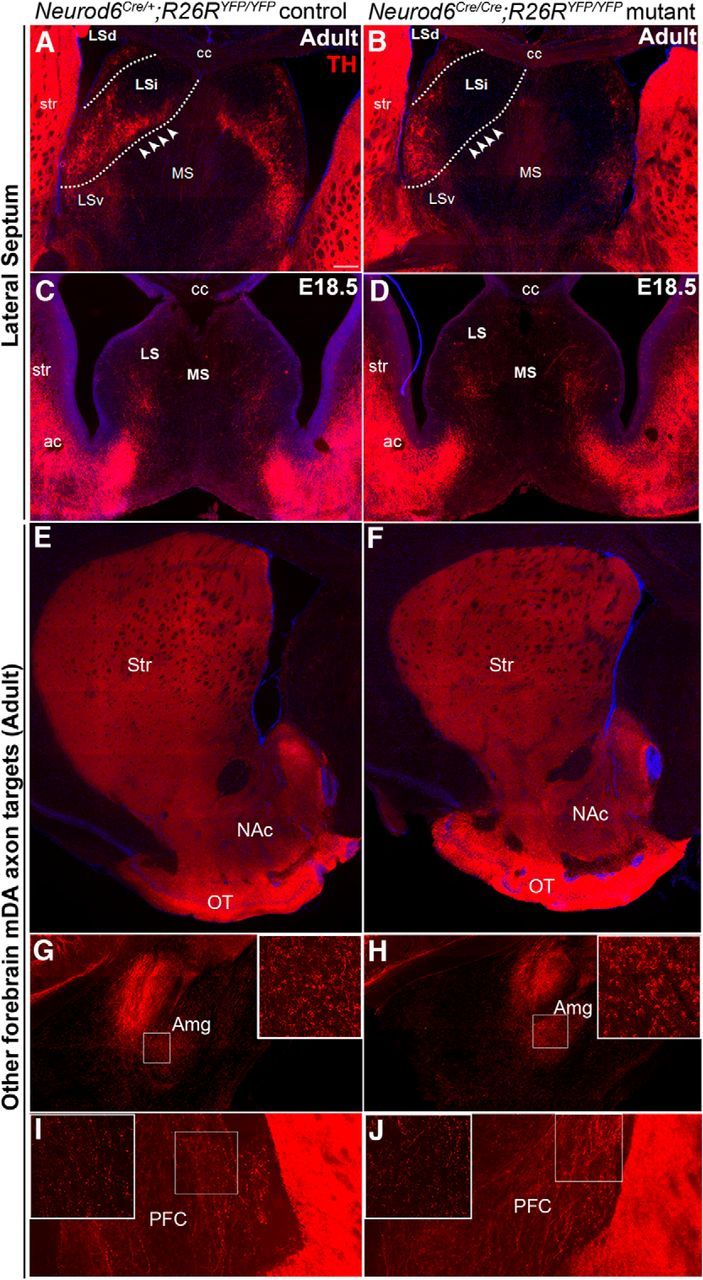
TH^+^ mDA axon projections to the intermediate region of the lateral septum are specifically missing in adult *Neurod6* mutant mice. ***A***, ***B***, TH immunohistochemistry shows specific loss of axon projections (arrowheads) to the LSi but not to LSd in *Neurod6* mutant adult mice. ***C***, ***D***, TH^+^ axon projections from mDA neurons have not yet reached the dorsal region of the lateral septum (LS) at E18.5. ***E–J***, TH^+^ mDA neuronal projections to other target sites of the VTA, including nucleus accumbens (NAc), olfactory tubercle (OT), amygdala (Amg), and prefrontal cortex (PFC), appear normal in *Neurod6* mutant compared with control adult mice. TH^+^ axons of SNc mDA neuronal projections to the striatum (Str) were also unaffected. MS, Medial septum; LSv, lateral septum, ventral; cc, corpus callosum; ac, anterior commissure. ***G–J***, Insets, Higher magnification of boxed region. Scale bars: ***A***, 200 μm.

The loss of a subset of VTA neurons in *Neurod6* mutant mice is likely to affect the connectivity between the VTA and other brain regions. To address this possibility, we first compared the axon projections of mDA neurons in adult *Neurod6* mutant and control mice by immunohistochemistry for TH. We analyzed the neuronal projection targets of VTA neurons (A10) in the septum, prefrontal cortex, nucleus accumbens, amygdala, and olfactory tubercle. In control mice, a dense arborization of TH^+^ axons was observed in the LSi, and fine varicosities of TH^+^ mDA axons were found in the LSd (Fig. 5*A*,*B*). The dense arbor in the LSi was completely absent in *Neurod6* mutants, whereas the axons of the LSd were unaffected (Fig. 5*A,B*). The specific loss of TH^+^ mDA axons in the LSi was also observed at P3, P7, and P14 (data not shown). We also examined the septal target site at E18.5 before the loss of YFP^+^ mDA neurons in mutant embryos; however, TH^+^ fibers were not detected in the dorsal lateral septal areas of control embryos, suggesting that at this stage TH^+^ mDA axons have not yet reached this target site (Fig. 5*C,D*). Consequently, it was not possible to analyze axon targeting before cell death in *Neurod6* mutants. In contrast to the defects observed in the lateral septum, TH^+^ mDA axons in all other A10 neuronal target sites also appeared normal in *Neurod6* mutant mice (Fig. 5*E–J*). The loss of both *Neurod6*^+^ neurons and TH^+^ axonal projections in *Neurod6* mutant mice suggests that *Neurod6*^+^ VTA neurons project to the intermediate regions of the lateral septum.

### FG retrograde labeling demonstrates that *Neurod6*^+^ mDA neurons project to the lateral septum

**Figure 6.**
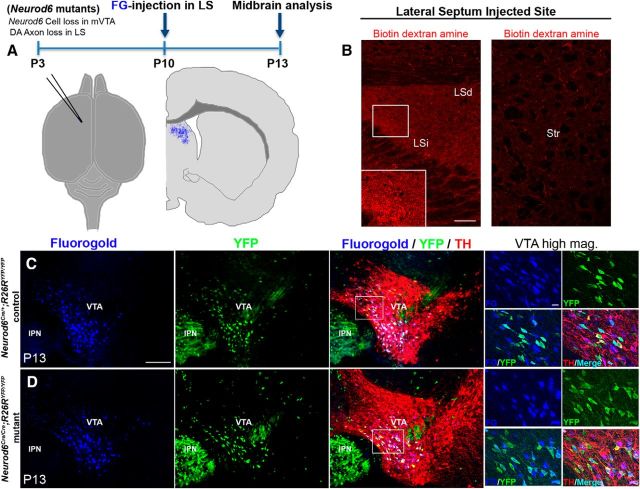
FG retrograde labeling experiments show that *Neurod6*^+^ mDA neurons project to the dorsal and intermediate region of the lateral septum. ***A***, Schematic diagram indicating the position of FG injection in the injected brain and the schedule of the experiment. ***B***, BDA is detected specifically in the LSi as well as the LSd but not in the adjacent striatal regions. ***C***, ***D***, Injection of FG into the septal region results in its retrograde transport of FG into YFP^+^/TH^+^ mDA cell bodies in *Neurod6* control and mutant mice by P13. Small panels indicate higher magnification of the corresponding boxed regions. IPN, interpeduncular nucleus; mag, magnification. Scale bars: ***B***, 100 μm; ***C***, 200 μm (20 μm, higher magnification).

To determine whether the septum is a specific target site for *Neurod6*^+^ mDA neurons, we next performed FG retrograde labeling experiments. FG was injected into the lateral septum of *Neurod6* control and mutant pups at P10, and retrograde transport of FG into cell bodies of mDA neurons was analyzed at P13 (Fig. 6*A*). We first confirmed that FG had been specifically injected into the LSd and LSi regions of the septum by staining for BDA, which was coinjected with the FG. Coronal sections of the forebrain at the level of the septum showed BDA^+^ fibers in the lateral septal region, but not in the adjacent striatal region (Fig. 6*B*). In the midbrain of *Neurod6*^+^ control mice at P13, FG labeled most YFP^+^/TH^+^ mDA neurons in the IF, dPN, vPN, and PBP of the VTA, demonstrating that *Neurod6*^+^ mDA neurons are lateral septal-projecting neurons (Fig. 6*C*). In *Neurod6* mutant mice, intraseptal FG injections retrogradely labeled many of the YFP^+^/TH^+^ mDA neurons that remain in the vPN and PBP of the VTA, indicating that these neurons project to the LSd and their axons likely correspond to the remaining TH^+^ fibers in the LSd of *Neurod6* mutants (Fig. 6*D*). Together, these results suggest that the YFP^+^/TH^+^ mDA neurons missing in *Neurod6* mutant animals project to the LSi, whereas the remaining YFP^+^/TH^+^ cells in these mutants project to the LSd. In addition, some YFP^−^/TH^+^ mDA neurons are labeled by FG, indicating that *Neurod6*^−^ mDA neurons also project to the lateral septum (Fig. 6*C,D*).

### Severe loss of *Neurod6*^+^ VTA neurons in Neurod1 and Neurod6 double mutants

**Figure 7.**
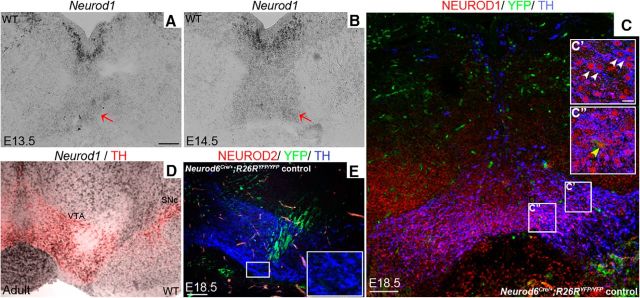
*Neurod1*, but not *Neurod2*, is expressed in mDA neurons. ***A***, ***B***, *In situ* hybridization shows that *Neurod1* transcripts are expressed strongly and weakly, respectively, in immature and mature (red arrows) in mDA neurons located ventral to the floor plate of the midbrain at E13.5 (***A***) and E14.5 (***B***). ***C–E***, This expression, detected by triple immunolabeling with NEUROD1, TH, and YFP antibodies, is maintained in mature YFP^+^ and TH^+^ mDA neurons at E18.5 (***C–C″***) and in adult (***D***) *Neurod6* control mice by *in situ* hybridization of *Neurod1* followed by immunohistochemistry of GFP and TH. ***E***, In contrast, NEUROD2 was not detected in mDA neurons at E18.5 by immunohistochemistry in sections of central mDA regions. ***C′***, ***C″***, ***D***, Insets, Higher magnification of corresponding boxed areas. ***C′***, White arrowheads indicate double-labeled cells. ***C″***, Yellow arrowhead indicates triple-labeled cells. Scale bars: ***A***, ***D***, 100 μm; ***C′***, 10 μm; ***E***, 100 μm.

Because *Neurod* family members share redundant roles in other parts of the nervous system, we next addressed the possibility that another *Neurod* gene promotes the survival of a subset of *Neurod6*^+^ neurons in *Neurod6* mutants. We first examined the expression profiles of NEUROD1 and NEUROD2 in mDA neurons. *Neurod1* transcripts were detected by *in situ* hybridization strongly in immature and weakly in mature mDA neurons at E13.5 and E14.5 (Fig. 7*A*,*B*). NEUROD1 expression analyzed by immunohistochemistry of *Neurod6*^*Cre*/+;^*Rosa26^YFP/YFP^* embryos was maintained in all mature TH^+^ mDA neurons, including YFP^+^ (*Neurod6*^+^) mDA neurons at E18.5. In addition, *in situ* hybridization of *Neurod1* followed by immunohistochemistry of TH showed that this expression was maintained into the adult stage (Fig. 7*D*). In contrast, NEUROD2 analyzed by immunohistochemistry was not expressed in mDA neurons at E18.5 (Fig. 7*D*).

**Figure 8.**
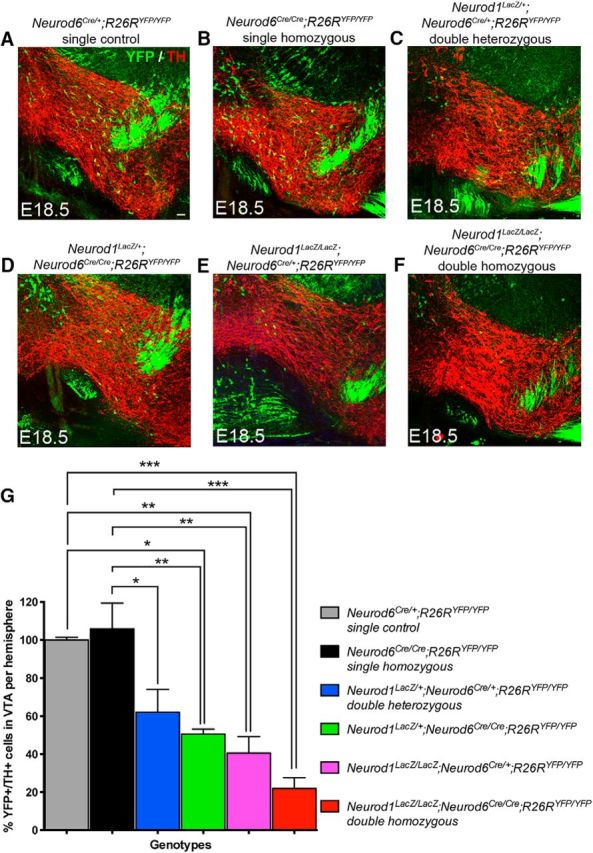
*Neurod1* is also required for the survival of *Neurod6*^+^ mDA neurons. ***A–F***, YFP^+^/TH^+^ mDA neurons are lost at E18.5 in *Neurod1;Neurod6* double mutants carrying different copy numbers of *Neurod1* and *Neurod6* mutant alleles. Immunohistochemistry of YFP and TH shows that reduced numbers of YFP^+^/TH^+^ mDA neurons are observed in coronal sections of the central midbrain in double-mutant embryos carrying 2 (***C***), 3 (***D***, ***E***), or 4 *Neurod1;Neurod6* mutant alleles (***F***). In contrast, *Neurod6* single-homozygous mutants present no change in YFP^+^/TH^+^ expression in mDA neurons at E18.5 (***B***). ***G***, Bar graph represents percentage changes of the number of YFP^+^/TH^+^ mDA neurons in the central mDA region of *Neurod1;Neurod6* double and *Neurod6* single-mutant embryos normalized to *Neurod6*^*Cre*/+^ control embryos. Error bars indicate SEM; *N* = 3 per genotype. *p < 0.05 (one-way ANOVA, with Tukey's post test). **p < 0.01 (one-way ANOVA, with Tukey's post test). ***p < 0.0001 (one-way ANOVA, with Tukey's post test). Scale bars: ***A***, 100 μm.

To determine whether *Neurod1* is required for the survival of *Neurod6*^+^ mDA neurons, we used mice carrying a null allele of *Neurod1* whereby *LacZ* replaces the *Neurod1* coding sequence (Naya et al., 1997; Miyata et al., 1999) to generate *Neurod1* and *Neurod6* (*Neurod1;Neurod6*) double-mutant mice (*Neurod1^LacZ/LacZ^;Neurod6^Cre/Cre^;R26R*). The double-homozygous mutants died shortly after birth at P0, as the *Neurod1^LacZ/LacZ^* single mutants that die from neonatal diabetes (Miyata et al., 1999). We therefore examined *Neurod6*^+^ VTA neurons at E18.5 and observed a severe loss of YFP^+^/TH^+^ mDA neurons in the VTA of *Neurod1;Neurod6* double-homozygous mutants (Fig. 8*F*,*G*) compared with both control (Fig. 8*A*,*G*) and *Neurod6^Cre/Cre^* single-mutant embryos (Fig. 8*B*,*G*), which do not show a cell loss at this stage. Moreover, mice homozygous mutant for *Neurod1* and heterozygous for *Neurod6* (*Neurod1^LacZ/LacZ^;Neurod6*^*Cre*/+^) as well as mice homozygous mutant for *Neurod6* and heterozygous for *Neurod1* (*Neurod1*^*LacZ*/+^*;Neurod6^Cre/Cre^*) and double heterozygous mice (*Neurod1*^*LacZ*/+^*;Neurod6*^*Cre*/+^ mice) also present significant losses of YFP^+^/TH^+^ mDA neurons compared with *Neurod6^Cre/Cre^* single mutants at E18.5 (Fig. 8*D–F*, respectively, and Fig. 8*G* and Table 2). These results suggest that both *Neurod1* and *Neurod6* contribute to the survival of VTA *Neurod6*^+^ neurons before birth because loss of one copy of each gene reduces neuronal viability. They also suggest that *Neurod1* has a more important role than *Neurod6* in *Neurod6*^+^ neuron survival because loss of one copy of *Neurod1* and one copy of *Neurod6* results in the loss of some neurons, whereas loss of two copies of *Neurod6* has no effect at E18.5. However, the early postnatal death of double-mutant mice precluded the analysis of mDA neurons and axonal projections at later stages. Earlier analysis of double-mutant embryos was also not feasible because YFP, which serves to mark *Neurod6*^+^ mDA neurons, only becomes detectable at E18.5 (data not shown).

**Table 2.** Total numbers of YFP^+^/TH^+^ cells counted in the central mDA region of *Neurod6* and *Neurod1* double mutants per brain hemisphere at E18.5*^a^*

Genotype	*n*	Mean ± SEM
*Neurod6*^*Cre/*+;^*R26R*^*YFP/YFP*^ single controls	3	200 ± 3
*Neurod6*^*Cre/Cre*^*;R26R*^*YFP/YFP*^ single homozygous	3	211 ± 27
*Neurod1*^*LacZ/*+^*;Neurod6*^*Cre/*+;^*R26R*^*YFP/YFP*^ double heterozygous	3	124 ± 24
*Neurod1*^*LacZ/*+^*;Neurod6*^*Cre/Cre*^*;R26R*^*YFP/YFP*^	3	101 ± 5
*Neurod1*^*LacZ/LacZ*^*;Neurod6*^*Cre/*+^*;R26R*^*YFP/YFP*^	3	81 ± 17
*Neurod1*^*LacZ/LacZ*^*;Neurod6*^*Cre/Cre*^*;R26R*^*YFP/YFP*^ double homozygous	3	44 ± 11

*^a^*Raw data of the numbers of mDA neurons counted per brain hemisphere in the central mDA region among *Neurod6* single, *Neurod1;Neurod6* double mutant, and control brains at E18.5. *n*, number of brain samples analyzed.

## Discussion

In this paper, we have identified a novel subset of mDA neurons in the VTA that are marked by expression of the bHLH transcription factor NEUROD6. *Neurod6*^+^ neurons express a combination of molecular markers, including OTX2, ALDH1A1, CALBINDIN1, and *Grp*, and project to the LSi and LSd regions of the forebrain. Genetic studies revealed that *Neurod6* alone is required for the survival of LSi-projecting *Neurod6*^+^ mDA neurons postnatally, whereas the survival of *Neurod6*^+^ mDA neurons is dependent on both *Neurod1* and *Neurod6* embryonically. These results have identified a novel subset of mDA neurons projecting to the lateral septum and revealed essential roles for NEUROD-family proteins in regulating the survival of this mDA neuronal subset. Our molecular characterization of this population of neurons will also facilitate further genetic manipulations to study the functions of these septal-projecting mDA neurons.

### *NeuroD6* identifies a novel subset of VTA mDA neurons that project to the lateral septum

Despite increasing evidence of heterogeneity among mDA neurons, there is still a paucity of specific markers to identify distinct mDA neuron subsets. mDA neurons in the VTA have been subdivided into molecularly distinct subsets based on combinational expression of more broadly expressed genes (Di Salvio et al., 2010a). For example, OTX2, CALBINDIN1, and ALDH1A1 mark a ventral subset of VTA neurons, whereas OTX2^+^/CALBINDIN1^+^ and OTX^+^/GIRK2^+^ label central and medial VTA neurons, respectively. Our molecular characterization shows that VTA mDA neurons can be further subdivided by the expression of *Neurod6*. Recent studies using single-cell transcriptome profiling have also identified a molecularly distinct subset of mDA neurons in the VTA that coexpresses *Otx2*, *Aldh1a1*, *Calb*, *Grp*, *Lpl*, and *Adcyap1* (Poulin et al., 2014). Because *Neurod6*^+^ mDA neurons also express the first four of these markers, it is probable that *Neurod6* marks the same subset. *Neurod6* is however uniquely expressed in this neuronal subset, in contrast to OTX2, CALBINDIN1, and ALDH1A1, which all have a broader expression in mDA neurons (Di Salvio et al., 2010a; Poulin et al., 2014). Our anatomical studies also showed that *Neurod6*^+^ mDA neurons have a complex spatial organization and are distributed in the PB, PN, and IF nuclei of the adult VTA.

We performed FG retrograde tracing experiments, which established that *Neurod6*^+^ neurons project to the septum. In this region, *Neurod6*^+^ mDA neurons project to both the LSi and LSd. Loss of TH^+^ fibers in the LSi of *Neurod6* mutants suggests that LSi is uniquely targeted by *Neurod6*^+^ mDA neurons. In contrast, the remaining TH^+^ fibers in the LSd likely correspond to axon termini of both *Neurod6*^+^ and *Neurod6*^−^ mDA neurons because both populations were labeled by FG in the retrograde labeling experiments. As VTA axonal projections are thought to be mainly unbranched (Yetnikoff et al., 2014), we have not determined whether *Neurod6*^+^ mDA neurons also project to other forebrain regions. However, recent studies using single-cell transfection with viral vectors have suggested the existence of different subgroups of VTA neurons targeting one or more forebrain structures (Aransay et al., 2015). *Neurod6* is also expressed in the interpeduncular nucleus and TH^−^ cells in the rostral linear nucleus of raphe (Fig. 3*B*,*C*); hence, it is difficult to specifically trace the axon projections of *Neurod6*^+^/TH^+^ mDA neurons using stereotaxic viral injections. Instead, we plan to use a dual recombinase intersectional genetic strategy (for review, see Dymecki and Kim, 2007) to determine the full projection target(s) of *Neurod6*^+^*/*TH^+^ mDA neurons in future experiments.

Consistent with an earlier report in the rat, our results showed that TH^+^ axon projections to the septum become established at early postnatal stages because the TH^+^ fibers were first observed in P3 pups and not in E18.5 embryos (Antonopoulos et al., 1997). The role of dopamine in the lateral septum is poorly studied. Lesions of septal dopaminergic terminals by injection of 6-hydroxydopamine into the lateral septum of rats result in deficits in spatial memory tasks (Simon et al., 1986). Our molecular and neuroanatomical characterization of *Neurod6*^+^ septal-projecting mDA neurons, including their expression of the neuropeptide Grp that is implicated in the regulation of memory associated with fear and emotional arousal, social interaction, and food intake (for review, see Roesler and Schwartsmann, 2012), will aid in classifying these neurons and studying their potential functions in regulating memory and emotional behaviors.

### *Neurod6* and *Neurod1* regulate the survival of septal-projecting mDA neurons

Our analysis revealed a 30% reduction in the number of *Neurod6*^+^ mDA neurons in the VTA of *Neurod6* single-mutant mice at P3, and this phenotype was maintained in adult mutant mice. Loss of these neurons was accompanied by loss of TH^+^ fibers in the LSi of *Neurod6* single mutants, indicating that *Neurod6* alone is required for the survival of LSi-projecting mDA neurons. Because NEUROD1 is also expressed in both immature and mature mDA neurons during development and *Neurod6*^+^ mDA neurons are partially lost in *Neurod6* single mutants, we also analyzed the phenotypes of *Neurod1;Neurod6* double-mutant mice. Severe loss of *Neurod6*^+^ mDA neurons occurred in double-homozygous mice at E18.5 (i.e., before the cells are lost in *Neurod6* single mutants). *Neurod6*^+^ mDA neurons were also lost in double heterozygous mice and in mice homozygous for the *Neurod1* mutation and heterozygous for the *Neurod6* mutation. Because of the unavailability of NEUROD6-specific antibodies, we cannot determine the status of *Neurod6*^+^ mDA neurons in *Neurod1* single mutants. Together, our genetic analysis demonstrates that both *Neurod6* and *Neurod1* contribute to the survival of *Neurod6*^+^ mDA neurons. It also suggests that *Neurod1* has a more important role than *Neurod6* for *Neurod6*^+^ mDA neuronal survival at E18.5 because loss of the two copies of *Neurod6* has no effect, whereas loss of one copy of *Neurod1* and one copy of *Neurod6* reduces survival of these neurons before birth.

NEUROD6 has been shown to have a neuroprotective role in PC12 cells serum deprived or treated with the mitochondrial stressor rotenone, and to act by enhancing mitochondrial biogenesis (Uittenbogaard et al., 2010; Baxter et al., 2012). Consistent with these findings, we observed a decrease in mitochondrial mass before the death of *Neurod6* mutant mDA neurons, suggesting that defects in energy metabolism contribute to the apoptosis of these neurons. Support for this hypothesis comes from an earlier study indicating that NEUROD6 maintains the expression of nuclear-encoded mitochondrial factors known to regulate mitochondrial biogenesis in PC12 cells (Uittenbogaard and Chiaramello, 2005). Alternatively or in addition, changes in mitochondrial mass might be an indirect consequence of neurons undergoing apoptosis because *Neurod6* may also regulate the expression of trophic factors, such as brain-derived growth factor, as has been shown for *Neurod2* (Olson et al., 2001). The latter mechanism could explain why surrounding *Neurod6*^−^ mDA neurons also exhibit a decrease in mitochondrial mass. Further studies will be required to identify the mechanisms through which NEUROD family proteins regulate the survival of embryonic mDA neurons during development and to determine whether these proteins continue to have a survival role in these neurons during adult life.
